# Kernel of Corn in the Trachea

**Published:** 1867-02

**Authors:** E. Andrews

**Affiliations:** Prof. of Surgery in Chicago Medical College; Chicago


ARTICLE X.

### KERNEL OF CORN IN THE TRACHEA.—TRACHEOTOMY.—EXPULSION OF FOREIGN BODY.

By E. ANDREWS, M.D., Prof. of Surgery in Chicago Medical College.

Mr. S., of the town of M., brought me his child, which had, a few days previously, been playing with some corn-cobs, and had been seized with a sudden fit of strangling and coughing. The child was feverish, coughed constantly, had little sleep, and showed a distressed and anxious expression of countenance. The foreign body could be distinctly heard to rattle up and down the trachea with every inspiration and expiration. I administered ether to anæsthesia, and cut down upon the trachea above the isthmus of the thyroid body. After clearing the surface of the organ so as to have a distinct view. I inserted a scalpel and severed three or four rings of the trachea, making a perpendicular slit from the thyroid body to the cricoid cartilage. Drawing asunder this opening the next expiration drove the kernal of corn up into the orifice, where it hung an instant and was then sucked in again and disappeared. The second expiration brought it up again and expelled it with such force that it flew two feet from the patient. The incison was then closed with adhesive straps, and the patient made a good recovery.

